# Distribution and Frequency of *kdr* Mutations within *Anopheles gambiae* s.l. Populations and First Report of the *Ace.1*G119S Mutation in *Anopheles arabiensis* from Burkina Faso (West Africa)

**DOI:** 10.1371/journal.pone.0101484

**Published:** 2014-07-31

**Authors:** Roch K. Dabiré, Moussa Namountougou, Abdoulaye Diabaté, Dieudonné D. Soma, Joseph Bado, Hyacinthe K. Toé, Chris Bass, Patrice Combary

**Affiliations:** 1 IRSS (Institut de Recherche en Sciences de la Santé), Centre Muraz, Bobo-Dioulasso, Burkina Faso; 2 Department of Vector Biology, Liverpool School of Tropical Medicine, Liverpool, United Kingdom; 3 Biological Chemistry and Crop Protection Rothamsted Research, Harpenden, United Kingdom; 4 National Malaria Control Programme, Ministry of Health, Ouagadougou, Burkina Faso; National Institute for Communicable Diseases/NHLS, South Africa

## Abstract

An entomological survey was carried out at 15 sites dispersed throughout the three eco-climatic regions of Burkina Faso (West Africa) in order to assess the current distribution and frequency of mutations that confer resistance to insecticides in *An. gambiae* s.l. populations in the country. Both knockdown (*kdr*) resistance mutation variants (L1014F and L1014S), that confer resistance to pyrethroid insecticides, were identified concomitant with the *ace-*1 G119S mutation confirming the presence of multiple resistance mechanisms in the *An. gambiae* complex in Burkina Faso. Compared to the last survey, the frequency of the L1014F *kdr* mutation appears to have remained largely stable and relatively high in all species. In contrast, the distribution and frequency of the L1014S mutation has increased significantly in *An. gambiae* s.l. across much of the country. Furthermore we report, for the first time, the identification of the *ace.1* G116S mutation in *An. arabiensis* populations collected at 8 sites. This mutation, which confers resistance to organophosphate and carbamate insecticides, has been reported previously only in the *An. gambiae* S and M molecular forms. This finding is significant as organophosphates and carbamates are used in indoor residual sprays (IRS) to control malaria vectors as complementary strategies to the use of pyrethroid impregnated bednets. The occurrence of the three target-site resistance mutations in both *An. gambiae* molecular forms and now *An. arabiensis* has significant implications for the control of malaria vector populations in Burkina Faso and for resistance management strategies based on the rotation of insecticides with different modes of action.

## Introduction

The pyrethroid class of insecticides have become a mainstay for vector control since the ban of DDT due to off-target toxicity and the development of resistance. They have been most widely used to treat bed nets (ITNs) dedicated to personal and community protection [1], [2], [3]. Unfortunately, knock down resistance (*kdr*) to pyrethroids, which also confers cross-resistance to DDT, was first reported in *Anopheles gambiae* populations from Côte d’Ivoire [4]. Resistance likely resulted from the earlier intensive use of DDT and selection from pyrethroid use in crop protection particularly in cotton areas [5], [6]. *kdr* was initially shown to result from a point mutation (L1014F) in the pyrethroid target protein the voltage-gated sodium channel [7]. Based on a simple PCR diagnostic developed in the first report of the *kdr* mutation [7] several studies have been carried out on the distribution and the frequency of this mechanism throughout Africa. Initial studies showed that L1014F *kdr* was most widely distributed in West African *An. gambiae s.l.* populations [6], [8], [9]. This mutation was observed initially in the S molecular form of *An. gambiae* s.s. reaching high frequency but was not found either in sympatric mosquitoes of the M molecular form or *An. arabiensis* populations [5]. This provided further evidence of reproductive barrier between the M and S molecular forms [10], [11] and the two molecular forms of *An. gambiae* s.s. were recently confirmed as two distinct species termed *Anopheles coluzzii* for the M form and *Anopheles gambiae* for the S form [12]. However, a few years after the initial finding of the *kdr* mutation in the S molecular form, this mutation was also reported in the M form from the littoral of Benin and Côte d’Ivoire [13]. In-depth investigations carried out later in these geographic regions confirmed that this phenomenon was frequently observed in littoral but was rare inland [11]. DNA sequencing of these mosquitoes suggested that the mutation emerged in the M form by genetic introgression from the S form [14], [15]. In contrast, the emergence of the Leu-Phe *kdr* mutation within *Anopheles arabiensis* resulted from a *de novo* mutation event [15]. An extensive monitoring program in Burkina Faso has revealed that the L1014F *kdr* mutation initially detected in low frequency in the *An. gambiae* M molecular form and *An. arabiensis* [11], [15] has spread throughout the country and is observed in mosquito populations at relatively high frequency [16], [17]. Recently the L1014S *kdr*, which initially predominated in East Africa [18], [19], was reported in West Africa, first in Benin and then Burkina Faso within *An. arabiensis* populations [20], [21]. More recently this mutation was reported in a small number of individuals of the M and S forms of *An. gambiae* in Burkina Faso [22]. Taken together these results provide fundamental insight into the evolutionary processes underlying resistance in *Anopheles gambiae s.l.* Furthermore from an applied perspective, the emergence of resistance has significant implications for vector control programmes, especially those focused on the use of ITNs/Long-Lasting Insecticidal Nets (LLINs) or indoor residual sprayings (IRS). Although LLINs had shown good control of certain pyrethroid resistant populations [23] reduced efficacy of treated nets against *An. gambiae* populations with *kdr* resistance has since been reported [24].

Other insecticides belonging to the organophosphate (OP) and carbamate (CM) classes have been investigated to be used in mosaic, or in combination, with pyrethroids for bednet impregnation [25]. In addition to the use of LLINs, bendiocarb was recently used in IRS applications in West Africa through the President’s Malaria Initiative (PMI) roadmap [26]. Initially described in *Culex* populations from Côte-d’Ivoire [27] reduced susceptibility to OPs and CMs was observed in *An. gambiae* populations in the North of Côte d’Ivoire and related to the domestic use of insecticide [28]. *An. gambiae* populations from Benin with resistance to the CM bendiocarb were reported after just three year of IRS use [29]. A common mechanism of resistance to OP and CM insecticides results from a single point mutation (termed *ace-1^R^*)in the target protein the acetylcholinesterase enzyme [30]. This mutation results in a glycine to serine replacement at amino acid position 119 and can be detected by a simple PCR-Restriction Fragment Length Polymorphism (RFLP) diagnostic [31]. This approach has been used to examine the frequency and distribution of this mutation in Burkina Faso where it was found predominately in the *An. gambiae* S form and in low frequency in the M form [9], [16], [32]. A recent study suggested that the mutation had introgressed from one form to the other but the precise origin of the introgression could not be determined due to the small sample size [33]. Since then, extensive country-wide surveys were performed in Burkina Faso from 2008 to 2010 and no case of *An. arabiensis* carrying this mutation was reported, although sample sizes for this species were sometimes small [16], [17].

However insecticide resistance may also occur by other physiological mechanisms such as metabolic detoxification through increased enzyme activities (monooxygenases, esterases, or glutathione S- transferases) [34], [35].

Burkina Faso is composed of three agro-climatic areas which exhibit different patterns of insecticide use especially in relation to crop protection. The present study provides an update on the distribution and the prevalence of the *kdr* L1014 and L1014S and*ace-1^R^* mutations in *An. gambiae* s.l. populations throughout the 13 health regions dispersed across these different agro-climatic areas. We report here, for the first time, the occurrence of the *ace-1^R^* mutation at remarkably high frequencies in *An. arabiensis*.

## Materials and Methods

### Study sites

Burkina Faso covers three ecological zones, the Sudan savannah zone in the south and west where rainfall is relatively heaviest (5–6 months), the arid savannah zone (Sudan-sahelian) which extends throughout much of the central part of the country and the aridland (Sahel) in the north. The northern part of the country has a dry season of 6–8 months. The varied ecological conditions are reflected in the different agricultural systems practiced throughout the country, from arable to pastoral lands. The western region constitutes the main cotton belt extending to the south where some new cotton areas have been cultivated since 1996. All ecological zones support the existence of *Anopheles* species that vector malaria and the disease is widespread throughout the country. Larvae were sampled from 15 sites dispersed throughout the three ecological zones (Table 1). The GPS coordinates were incorporated in Table 1.

**Table 1:** Distribution of *Anopheles gambiae s.l.* from 15 sites in Burkina Faso.

Study sites	Geographic references	Social environment	Climatic areas	Agricultural practices	Date of collection	*An. gambiae* s.l.	*An. gambiae*		*An. coluzzii*		*An. arabiensis*	
						N	n1	%	n2	%	n3	%
Gaoua	10°40′N; 3°15′W	sub-urban	Sudanian	cereals, cotton,old area	30/10/2012	43	39	90,69	1	32,33	3	6,98
Banfora	10°40′N; 3°15′W	sub-urban	Sudanian	cereals, cotton,old area	09/07/2012	30	24	80,00	6	20,00	0	0
Sindou	10°40′N; 3°15′W	rural	Sudanian	cotton,old area	01/10/2012	35	24	68,57	6	17,14	5	14,29
Orodara	10°40′N; 3°15′W	sub-urban	Sudanian	fruits, cotton,old area	23/19/2012	28	23	82,14	4	14,29	1	3,57
Dioulassoba	10°40′N; 3°15′W	traditional-urban	Sudanian	swamp	23/11/2012	29	4	13,79	5	17,24	20	68,97
Soumousso	10°40′N; 3°15′W	rural	Sudanian	cotton,old area	30/12/2012	30	20	66,67	3	10,00	7	23,33
Boromo	10°40′N; 3°15′W	sub-urban	Sudan-sahelian	cotton,old area	08/10/2012	33	16	48,48	0	0	17	51,52
Dédougou	10°40′N; 3°15′W	sub-urban	Sudan-sahelian	cotton,old area	06/10/2012	30	12	40,00	2	6,67	16	53,33
Koudougou	10°40′N; 3°15′W	urban	Sudan-sahelian	cotton,since 1996	07/11/2012	37	19	51,35	5	13,51	13	35,14
Nanoro	10°40′N; 3°15′W	rural	Sudan-sahelian	cereals	09/07/2012	32	4	12,50	24	75,00	4	12,50
Koupela	10°40′N; 3°15′W	sub-urban	Sudan-sahelian	cottonsince 1996	06/10/2012	30	14	46,67	8	26,67	8	26,67
Fada	10°40′N; 3°15′W	sub-urban	Sudan-sahelian	cottonsince 1996	25/08/2012	60	19	31,67	27	45,00	14	23,33
Kaya	10°40′N; 3°15′W	sub-urban	Sahelian	cereals,vegetables	03/10/2012	32	15	46,88	5	15,63	12	37,50
Ouahigouya	10°40′N; 3°15′W	sub-urban	Sahelian	cereals,vegetables	08/10/2012	31	20	64,52	10	32,26	1	3,23
Dori	10°40′N; 3°15′W	sub-urban	Sahelian	cereals,vegetables	01/10/2012	33	12	36,36	5	15,15	16	48,48

N: number total of mosquitoes.

n1: number of *An. gambiae.*

n2: number of *An. coluzzii.*

n3: number of *An. arabiensis.*

### Mosquito sampling

Larvae of *An. gambiae* s.l. were collected from at least 10 breeding sites dispersed throughout each sampling site mainly comprising pools of standing water and other small water collections. Larvae were pooled to constitute a colony, which was reared in the insectary to adulthood. A sample of 100 adult females were randomly sorted, killed and kept on silica gel in 1.5-ml tubes and stored at −20°C prior to PCR analysis. Anopheline species were identified morphologically using the standard identification keys of Gillies and Cootzee [36].

### PCR analyses

An average of 30 mosquitoes was sampled per site by PCR analysis. Genomic DNA was extracted from single specimens and used as template for PCR to determine the species within the *An. gambiae* complex using the protocol SINE 200 of Santalomazza *et al.* [37] that allows the concomitant identification of *An. gambiae* M and S (respectively known as *Anopheles coluzzii* and *Anopheles gambiae*) and *An. arabiensis.* The same individuals were then tested for both the L1014F and L1014S *kdr* mutations using the protocols of Martinez-Torres *et al.* [7] (using specific primers Agd1, Agd2, Agd3 and Agd4) and Ranson *et al.* [18] (using Agd1, Agd2, Agd4 and Agd5) respectively:

- Agd1: 5′-ATAGATTCCCCGACCATG-3′;
- Agd2: 5′-AGACAAGGATGATGAACC-3′;
- Agd3: 5′-AATTTGCATTACTTACGACA-3′;
- Agd4: 5′-CTGTAGTGATAGGAAATTTA-5′;
- Agd5: 5′-TTTGCATTACTTACGACTG-3′.

The *ace-1^R^* mutation was detected from the same samples by PCR according to the protocol of Weill *et al*. [31] using specific primers *Ex3AGdir* (GATCGTGGACACCGTGTTCG) and *Ex3AGrev* (AGGATGGCCCGCTGGAACAG). Then the PCR products were digested using *Alu 1* enzyme at 37°C for 3 hours.

### Statistical analysis

Data were compared between ecological zones and pooled for each species to compare the genotypes frequency between *An. gambiae* species by Chi^2^ tests. The genotypic frequencies of L1014F and L1014S and *ace-1^R^* in mosquito populations were compared to Hardy-Weinberg expectations using the exact test procedures implemented in GenePOP (ver.3.4) software [38].

### Ethical issues

Ethical approval was not required in this study.

This study was not carried out on private land. For each, no permission was required our study does not degrade the environment. No permission was required for these locations/activities as the field activities did not involve damaged of protected species. We did not use any vertebrate during this study.

## Results

Out of 516 mosquitoes analysed in PCR, 513 successfully scored (less than 5% failure rate). Overall species composition of the collected mosquitoes comprised a higher proportion of *An. gambiae* (51.7%) than *An. coluzzii* (21.6%) and *An. arabiensis* (26.7%) (Table 1). The species repartition across the three ecological regions revealed that *An. gambiae* was the predominant species in all regions including, in the Sahel where it comprised more than 49% of the *An. gambiae s.l.* population. *Anopheles arabiensis* was the second most predominant vector found in samples collected from the three regions. Somewhat *An. coluzzii* was found at a relatively low proportion of less than 15%. The central areas were characterised by an overlapped repartition of the three species 38.4%, 27.81% and 33.75% for *An. gambiae, An. coluzzii* and *An. arabiensis* respectively and proportions did not differ significantly (χ^2^ = 1.95, df = 1, *P*>0.05). In the Sahel region, *An. gambiae* also predominated (49.75%) and the proportions of the two other species did not differ significantly at 21.01% and 29.74% for *An. coluzzii* and *An. arabiensis* respectively (χ^2^ = 4.88, df = 1, *P*>0.05).

**Figure 1:**
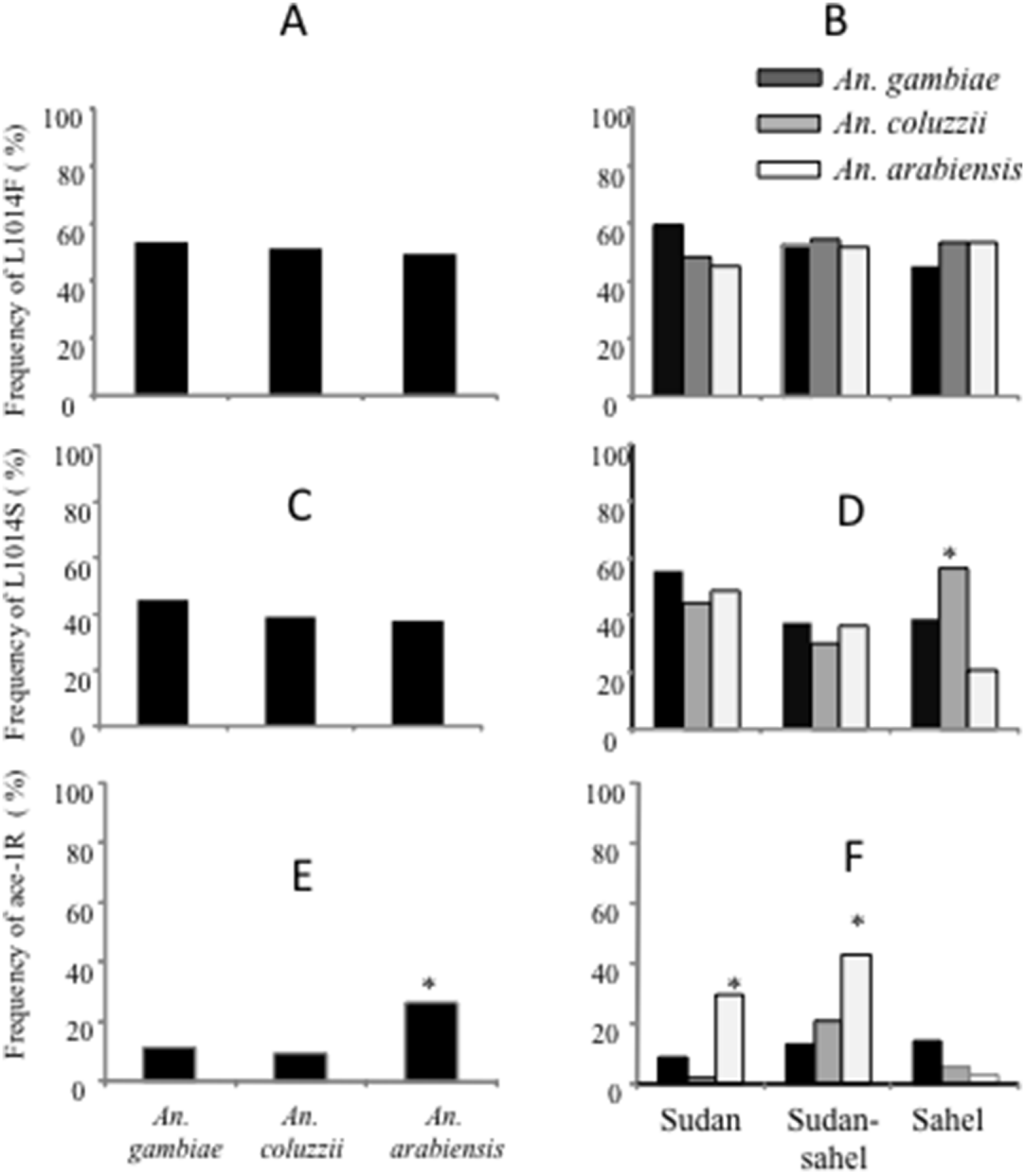
Comparison of allele frequencies of 1014F, 1014S and *ace-1^R^* mutations within *Anopheles gambiae*, *An. coluzzii* and *An. arabiensis* populations from 15 sites dispersed across the 3 agro-ecological regions of Burkina Faso.

**Figure 2:**
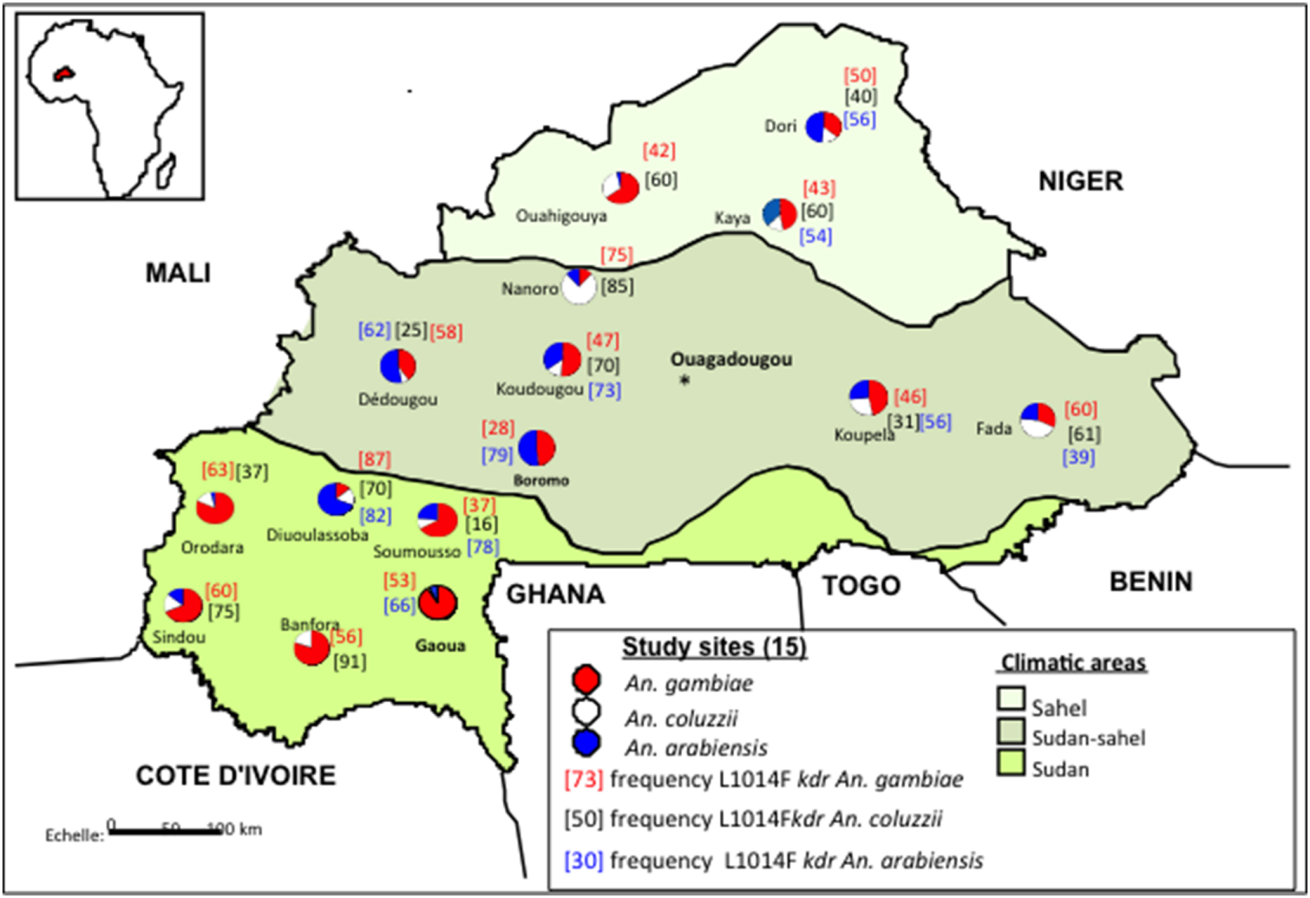
Distribution the 1014F *kdr* allele frequency from 15 sites dispersed across Burkina Faso.

The overall frequency of the L1014F mutation averaged 50% and did not significantly differ between species (Figure 1A) whatever the ecological zone (Figure 1B) (χ^2^ = 0.14, df = 1, *P*>0.05) even though the highest values were observed in the sudan zone (Figure 2). However some deviation from Hardy-Weinberg expectations was observed within the *An. arabiensis* populations in Dedougou and Dori and within *An. coluzzii* populations in Fada, Kaya, Ouahigouya and Dori with an excess of resistant homozygous alleles (Table 2). The same patterns were found in seven sites for *An. gambiae* (Gaoua, Banfora, Sindou in the West, Dedougou, Koudougou and Koupela in the central region and Ouahigouya in the Sahel) (*P*<0.05).

**Table 2:** Allelic and genotypic frequencies at the *kdr* 1014F and 1014S locus in *An. gambiae s.l* populations.

Species	Sites	N	Genotypes						Genotypes				
			1014L 1014L	1014L 1014F	1014F 1014F	f(L1014F)	[95%Cl]	p(HW)	1014L 1014L	1014L 1014F	f(L1014F)	[95%Cl]	p(HW)
***An. arabiensis***	Gaoua	5	1	0	2	0.66	[8.5–9.82]	-	0	2	0.66	[8.5–9.82]	0.2000
	Banfora	0	0	0	0	-	-	-	0	0	0.9	-	-
	Sindou	10	5	0	0	0	-	-	1	4	0	[7.38–9.18]	-
	Orodara	1	1	0	0	0	-	0.4678	0	0	0.45	-	-
	Dioulassoba	30	1	5	14	0.82	[3.13–4.71]	0.2308	2	8	0.42	[2.34–3.38]	0.0003
	Soumousso	11	1	1	5	0.78	[5.74–7.3]	0.0956	2	2	0.37	[4.37–5.21]	0.2914
	Boromo	17	2	3	12	0.79	[3.42–5.00]	0.000	6	3	0.28	[2.31–3.35]	0.3405
	Dédougou	23	6	0	10	0.62	[3.23–4.47]	0.1652	5	2	0	-	0.3213
	Koudougou	13	2	3	8	0.73	[3.9–5.36]	-	0	0	0.5	[6.41–7.41]	-
	Nanoro	6	4	0	0	0	-	0.4406	0	2	0.5	[4.39–5.39]	0.0857
	Koupela	13	2	3	3	0.56	[4.61–5.73]	0.2970	2	3	0.53	[4.39–5.39]	0.1795
	Fada	25	6	5	3	0.39	[2.87–3.65]	0.0933	8	3	0.57	[3.42–4.48]	0.9035
	Kaya	17	4	3	5	0.54	[3.61–4.69]	-	1	4	0.37	[3.07–3.81]	0.0061
	Ouahigouya	1	0	1	0	0.5	[3.32–4.32]	0.0031	0	0	0	[18.5–20.5]	-
	Dori	22	6	2	8	0.56	[3.1–4.22]	-	4	2	0.26	[2.32–2.84]	0.2260
***An. coluzzii***	Gaoua	1	1	0	0	0	[18.5–20.5]	-	0	0	0	[18.5–20.5]	-
	Banfora	7	0	1	5	0.91	[6.69–8.51]	-	0	1	0.16	[3.04–3.36]	0.0909
	Sindou	12	1	1	4	0.75	[6.15−/.65]	0.2727	1	5	0.91	[6.69–8.51]	-
	Orodara	5	2	1	1	0.37	5.58–6.32]	0.4286	1	0	0.12	[3.27–3.51]	-
	Dioulassoba	9	1	1	3	0.7	[6.61–8.01]	0.3333	1	3	0.7	[6.61–8.01]	0.3333
	Soumousso	4	2	1	0	0.16	[4.36–4.68]	-	0	1	0.33	[6.16–6.82]	0.2000
	Boromo	0	0	0	0	-	-	-	0	0	-	-	-
	Dédougou	3	1	1	0	0.25	[6.67–7.17]	-	0	1	0.5	[9.28–10.28)	0.6190
	Koudougou	7	0	3	2	0.7	|6.6–8.01]	1	2	0	0.2	[3.72–4.12]	-
	Nanoro	39	1	5	18	0.85	[2.82–4.52]	0.3983	12	3	0.37	[2.06–2.8]	0.3333
	Koupela	9	3	5	0	0.31	|3.54–4.16]	1	1	0	0.06	[1.64–1.76]	0.7446
	Fada	46	7	7	13	0.61	[2.33–3.55]	0.0186	17	2	0.38	[1.94–2.7]	0.0817
	Kaya	8	2	0	3	0.6	[6.17–7.37]	0.0476	2	1	0.4	[5.13–5.93]	0.3333
	Ouahigouya	17	4	0	6	0.6	[4.19–5.39]	0.0017	2	5	0.6	[4.19–5.39]	1
	Dori	9	3	0	2	0.4	[5.13–5.93]	0.0476	1	3	0.7	[6.61–8.01]	-
***An. gambiae***	Gaoua	74	14	8	17	0.53	[3.75–2.81]	0.0002	0	35	0.92	[2.12–3.96]	1
	Banfora	29	7	7	10	0.56	2.43–3.55]	0.0434	3	2	0.14	[1.36–1.64]	0.1518
	Sindou	46	8	3	13	0.6	[2.49–3.69]	0.0003	5	17	0.81	[2.78–4.4]	0.0611
	Orodara	33	5	7	11	0.63	[2.6–3.86]	0.0904	1	9	0.41	[2.2–3.02]	0.0420
	Dioulassoba	8	0	1	3	0.87	[8.239.97]	-	2	2	0.75	[7.71–9.21]	0.3257
	Soumousso	29	8	9	3	0.37	[2.29–3.63]	0.5690	5	4	0.32	[2.16–2.8]	0.0000
	Boromo	25	8	7	1	0.28	|2.31–2.87]	0.7912	4	5	0.43	[2.78–3.64]	0.1201
	Dédougou	19	5	0	7	0.58	[3.72–4.88]	0.0004	7	0	0.29	[2.75–3.33]	0.0150
	Koudougou	26	9	2	8	0.47	[2.61–3.55]	0.0005	4	3	0.26	[2.03–2.55]	1
	Nanoro	5	1	0	3	0.75	[7.71–9.21]	0.1429	0	1	0.25	[4.64–5.14]	0.1429
	Koupela	24	7	1	6	0.46	[3.08–4.00]	0.0013	4	6	0.57	[3.37–4.51]	0.0003
	Fada	30	3	9	7	0.6	[2.87–4.07]	0.6254	5	6	0.44	[2.54–3.42]	0.0473
	Kaya	19	5	7	3	0.43	[2.88–3.74]	0.5785	3	1	0.16	[1.86–2.18]	0.0000
	Ouahigouya	30	10	3	7	0.42	[2.4–3.25]	0.0020	2	8	0.45	[2.48–3.38]	0.0632
	Dori	18	4	4	4	0.5	[3.49–4.49]	0.2300	1	5	0.55	[4.03–5.13]	0.0520

N: number of mosquitoes.

f(1014F): frequency of the kdr W resistant allele.

f(1014S): frequency of the kdr E resistant allele.

p(HW): probability of the exact test for goodness of fit to Hardy Weinberg equilibrium.

-: not determined.

**Figure 3:**
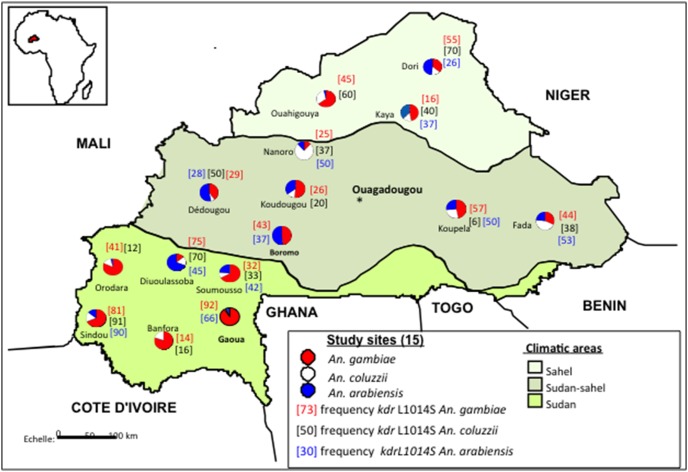
Distribution the 1014S *kdr* allele frequency from 15 sites dispersed across Burkina Faso.

The overall allele frequency of the L1014S *kdr* mutation (Figure 3) was relatively higher in *An. gambiae* (48%) followed by *An. coluzzii* (38%) and *An. arabiensis* populations (37%) with no significant difference between the last two (χ^2^ = 3.24, df = 1, *P*>0.05) (Figure 1C). Comparing between ecological regions, L1014S *kdr* frequency did not differ significantly between species, except in the Sahel where it was significantly higher in *An. coluzzii* than *An. arabiensis* (χ^2^ = 10.21, df = 1, *P*<0.001) and *An. gambiae* (*P*<0.04) (Figure 1D). The observed genotypic frequencies were not significantly different from Hardy-Weinberg expectations at the 95% confidence level (Table 2) in populations from any site except in the *An. gambiae* populations from Orodara, Soumousso, Koupela, Fada, and in the *An. arabiensis* populations from Dioulassoba and Kaya where a heterozygous deficit was observed (*P* = 0.005) and *An. gambiae* populations in two sites (Dedougou and Kaya) where an excess of heterozygotes was observed (*P*<0.05).

**Figure 4:**
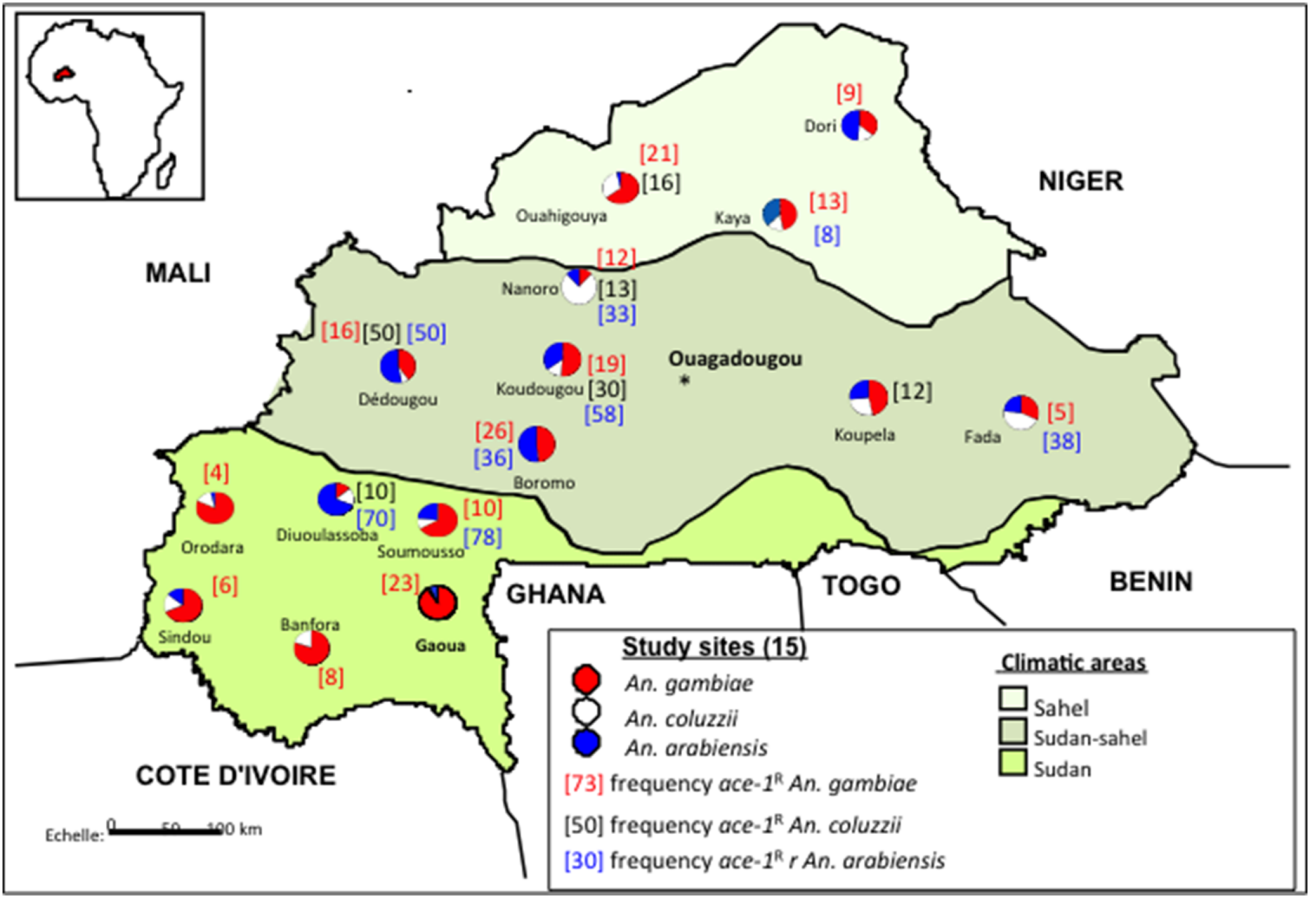
Distribution the *ace-1^R^* allele frequency from 15 sites dispersed across Burkina Faso.

The *ace-1^R^* mutation (Figure 4) was recorded in all the 15 sites under study with a wider distribution within the *An. gambiae* populations (Table 3). The overall allele frequency of *ace-1^R^* was significantly higher in *An. arabiensis* (0.26) than in *An. gambiae* (0.11) (χ^2^ = 14.4; df = 1, *P* = 0.001) and *An. coluzzii* (0.09) (χ^2^ = 11.77, df = 1, *P* = 0.006) (Figure 1E) with no significant difference between the last two (χ^2^ = 0.37, df = 1, *P* = 0.54). Compared between zones, the *ace-1^R^* allele frequency in *An. arabiensis* was higher than that of *An. coluzzii* (χ^2^ = 8.15, df = 1, *P* = 0.004) and *An. gambiae* (χ^2^ = 9.79, df = 1, *P*<0.001) in the Sudan and Sudan-sahelian savannah (with respectively χ^2^ = 6.89, df = 1, *P*<0.008 and χ^2^ = 17.34, df = 1, *P*<0.0003) (Fig. 1F). In the Sahel no significant difference was observed between the three species (χ^2^ = 0.89–0.021, df = 1, *P*>0.05). The observed genotypic frequencies were significantly different from Hardy-Weinberg expectations at the 95% confidence level (Table 3) in *An. gambiae* population from Orodara, Soumousso, Koudougou, Fada, Ouahigouya, Dori and Dioulassoba, Koudougou and Kaya for *An. arabiensis* where a heterozygote deficit was observed (*P* = 0.005). Furthermore, the percentage of homozygous resistant individuals was significantly higher in *An. arabiensis* (25%) than in *An. gambiae* (6.25%). No homozygous resistant individual was recorded in *An. coluzzii* from any site.

**Table 3:** Allelic and genotypic frequencies at the ace-1 locus in *An. gambiae s.l* populations from 15 sites in Burkina Faso.

Species	Sites	N	Genotypes			f(119S)	[95%Cl]	p(HW)
			119G 119G	119G 119S	119S 119S			
*An. arabiensis*	Gaoua	3	3	0	0	0	-	-
	Banfora	0	0	0	0	-	-	-
	Sindou	5	5	5	0	0	-	-
	Orodara	1	1	1	0	0	-	-
	Dioulassoba	20	4	4	12	0.7	[2.95–7.13]	0.0264
	Soumousso	7	1	1	5	0.78	[5.74–7.57]	0.2308
	Boromo	15	5	9	1	0.36	[2.67–5.42]	0.9488
	Dédougou	14	4	6	4	0.5	[3.19–7.25]	0.0444
	Koudougou	12	5	0	7	0.58	[3.72–9.1]	0.0004
	Nanoro	3	2	0	1	0.33	[6.16–17.45]	0.2000
	Koupela	8	8	0	0	0	-	-
	Fada	13	4	8	1	0.38	[2.96–6.26]	0.9449
	Kaya	12	11	0	1	0.08	[1.52–2.27]	0.0435
	Ouahigouya	1	1	0	0	0	-	-
	Dori	14	14	0	0	0	-	-
*An. coluzzii*	Gaoua	1	1	0	0	0	-	-
	Banfora	6	6	0	0	0	-	-
	Sindou	6	6	0	0	0	-	-
	Orodara	4	4	0	0	0	-	-
	Dioulassoba	5	4	1	0	0.1	[2.67–4.71]	-
	Soumousso	3	3	0	0	0	-	-
	Boromo	0	0	0	0	-	-	-
	Dédougou	2	0	0	2	0.5	[9.28–34.65]	1
	Koudougou	5	2	3	0	0.3	[4.49–10.78]	1
	Nanoro	23	17	6	0	0.13	[1.34–2.04]	1
	Koupela	8	6	2	0	012	[2.28–3.9]	1
	Fada	27	27	0	0	0	-	-
	Kaya	5	5	0	0	0	-	-
	Ouahigouya	9	6	3	0	0.16	[2.64–4.39]	1
	Dori	5	5	0	0	0	-	-
*An. gambiae*	Gaoua	36	22	11	3	0.23	[1.33–2.2]	0.2811
	Banfora	24	20	4	0	0.08	[1.05–1.46]	1
	Sindou	24	21	3	0	0.06	[0.92–1.23]	1
	Orodara	23	22	0	1	0.04	[0.74–0.99]	0.0222
	Dioulassoba	4	4	0	0	0	-	-
	Soumousso	20	18	0	2	0.1	[1.29–1.88]	0.0021
	Boromo	15	9	4	2	0.26	[2.32–4.31]	0.2260
	Dédougou	12	8	4	0	0.16	[2.1–3.59]	1
	Koudougou	18	14	1	3	0.19	[1.82–3.07]	0.0029-
	Nanoro	4	3	1	0	0.12	[3.27–6.29]	-
	Koupela	12	12	0	0	0	-	-
	Fada	19	18	0	1	0.05	[0.96–1.27]	0.0270
	Kaya	15	11	4	0	0.13	[1.69–2.62]	1
	Ouahigouya	19	14	2	3	0.21	[1.85–3.16]	0.0096
	Dori	11	10	0	1	0.09	[1.68–2.59]	0.0476

N: number of mosquitoes.

f(119S): frequency of the 119S resistant ace.1 allele.

p(HW): probability of the exact test for goodness of fit to Hardy Weinberg equilibrium.

-: not determined.

## Discussion

This study provides current information on the distribution of three members of the *Anopheles gambiae* complex across Benin and the frequency and distribution of three important target-site resistance mechanisms in these populations. In regards to the distribution of *An. gambiae* species throughout the country, the most significant finding is that *An*. *arabiensis* appears to be spreading in the Sudan whereas in the past it comprised only around 5% of the *An. gambiae* complex species [6]. Furthermore, this species is now present in Sindou at 14.29% (nearest the frontier of Cote-d’Ivoire) where it was absent a decade ago [9]. The reason for this is not clear but could be related to climatic changes, such as irregularities in rainfall observed in the boundaries of the Sudan region that may make the landscape more favourable to the establishment of this species.

Across sampling covering 15 sites we identified the L1014F and L1014S *kdr* mutations concomitant with the *ace-*1 G119S mutation confirming the presence of multiple resistance mechanisms in the *An. gambiae* complex in Burkina Faso [16], [17]. The distribution and the prevalence of the L1014F *kdr* mutation in *An. gambiae* species including *An. gambiae, An. coluzzii* and *An. arabiensis,* has been well documented in Burkina Faso for over a decade [9], [16]. Many studies reported this mutation at high frequency within *An. gambiae* and *An. coluzzii* populations especially in *An. gambiae* populations from the Sudan area where mutation frequency was approaching fixation [9], [15], [16]. Over recent years the frequency of this mutation has increased within both *An. coluzzii* and *An. arabiensis.* In this study although the L1014F mutation remains widespread in all three ecological regions and is present at relatively high frequency within the three species (averaging 50%), the frequencies reported in this current study were lower in the Sudan ecological regions (West and South West covering the old cotton belt) than those from previous studies [9], [16], [22]. For the other climatic zones i.e. central and northern regions the allele frequencies of L1014F varied within the three species with particularly high frequencies in *An. arabiensis*. The reason(s) for the reduction of L1014F frequency in *An. gambiae* populations in the Sudan area is not known, however, a similar trend was recently observed in the Western region of Burkina Faso where transgenic and biological control practices have been implemented for crop protection of cotton over the last four years (a long side conventional crop protection approaches) (Namountougou, unpublished). These alternative cotton-growing practices would be expected to reduce the quantity and frequency of insecticide use in agriculture and this may in turn reduce the selection pressure experienced by local mosquito populations. The analysis of observed genotypic frequencies revealed a heterozygote deficit for the L1014F mutation in the three species of *An. gambiae* s.l. from many sites especially in the Sahel for *An. coluzzii* and *An. arabiensis* and in the Sudan and Sudan-Sahel for *An. gambiae* which deviated significantly from Hardy-Weinberg expectations. This finding is not surprising as the same patterns were observed in the West (Orodara and Soumousso) four years ago [9] in combination with a novel mutation, N1575Y, in the voltage-gated sodium channel, recently reported in *An. gambiae* s.l. populations in Soumousso [39].

The L1014S *kdr* mutation was recently recorded at highest frequency in *An. arabiensis* populations in the centre on the country [21] and in Bobo-Dioulasso at frequencies averaging 38% [40]. Previous studies have recorded only a few individuals of *An. gambiae* and *An. coluzzii* from the Centre-East part of the country [17] carrying this mutation in the heterozygous form. The present study reveals that this mutation has since spread across the whole country and is now observed at relatively high and similar frequencies (40%) between the three species. The comparison of the observed genotypic frequencies of this mutation with that expected for Hardy-Weinberg equilibrium indicated, depending on the site, a deficit or excess of heterozygotes, mainly for *An. gambiae* populations. The occurrence of the L1014F *kdr* mutation in *An. coluzzii* had been suggested to have occurred by introgression from *An. gambiae* and via a *de novo* mutation event in *An. arabiensis* [15], however, the origin of the L1014S mutation in *An. gambiae*, *An. coluzzii* and *An. arabiensis* species in West Africa is not so clearly understood. The proximity of Burkina Faso from the Benin frontier where the L1014S mutation was first reported in *An. arabiensis* populations [20] suggests that it arrived in Burkina Faso via migration of *An. arabiensis* carrying the mutation from Benin, however, the origin of this mutation in *An. gambiae* and *An. coluzzii* populations in Burkina Faso remains to be elucidated.

In this study we report, for the first time, the presence of the *ace.1* G119S mutation in *An. arabiensis* populations from eight sites: Dioulassoba, Soumousso in the West, Boromo, Dédougou, Koudougou, Nanoro and Fada in the Centre-North and East and Kaya in the North. In these sites *An. arabiensis* was observed as the second major vector after *An. gambiae* except at Fada and Nanoro where the proportion of *An. arabiensis* was lower than that of *An. coluzzii*. To confirm this finding, we repeated the PCR amplification of *ace.1* ^R^ for our *An. arabiensis* specimens and used, as a control, 30 specimens of *An. Arabiensis* which we had confirmed in a previous study do not have this mutation. No false positives were observed in these samples suggesting our data is robust. The *ace.1* ^R^ allele was observed in this study in *An. arabiensis* at varying frequency reaching a maximum value of 78% in populations from Dioulassoba and the lowest value in Kaya at 8%. Except for samples from Soumousso and Nanoro where the sample size was not sufficient (n<10) to compare genotype frequencies, deviations from Hardy-Weinberg equilibrium were observed at three sites (Dioulassoba, Koudougou and Kaya) as a result of a high heterozygote deficit. The same pattern was observed in *An. gambiae* from Orodara, Soumousso, Koudougou, Fada, Ouahigouya and Dori. The deficit of heterozygous genotypes observed in Orodara and Soumousso is not new as Dabiré *et al.* [41] reported similar results from the these areas from which the duplicated allele (*ace.1* ^D^) was reported by Djogbenou *et al.* [33]. It is possible that this duplicated allele *ace.1^D^* is also present within *An. arabiensis* especially in Dioulassoba where the proportion of homozygous mutants was atypically high (60%). The high frequency of this mutation in Dioulassoba populations is intriguing as recent studies failed to find any L1014F *kdr* or *ace-1^R^* in *An. arabiensis* population from this site [40], [42]. As for the L1014S mutation, additional sequence analysis of the region flanking the *ace.1* locus are necessary to confirm whether the *ace.1* mutation in *An. arabiensis* has evolved along the same pathway as *kdr* e.g. as a *de novo* mutation or introgression from *An. gambiae* or *An. coluzzii*. Unfortunately our PCR data is not backed up by insecticide susceptibility bioassays and so we cannot assess the correlations between *kdr* and *ace*-1 mutations and the phenotypic expression of resistance.

The emergence of the *ace-1^R^* mutation in *An. gambiae* s.l. population from the cotton-growing areas may be linked to the agricultural use of OP and CM insecticides used for crop protection. Other sources of selection pressure outside the cotton belt include insecticide use for vegetable growing and domestic use of insecticide in public health. Bioassays performed in 2012 on *An. gambiae* populations from sites located in the cotton belt of the West of Burkina Faso revealed the development of resistance to CMs and OPs especially to benidocarb (Dabiré, unpublished) correlating with the prevalence and frequency of genetic resistance revealed in the present study. However, further bioassays on a wider scale are now required in order to understand the implications of the current status of the *ace-1^R^* mutation for the efficacy of OP and CM insecticides in vector control in Burkina Faso. The information provided by such studies combined with the genetic data presented here is a prerequisite for the informed use of CM and OP based-combinations for bednet impregnation and/or indoor residual spraying.
