# Correction: Distribution and Frequency of *kdr* Mutations within *Anopheles gambiae* s.l. Populations and First Report of the *Ace*.*1*G119S Mutation in *Anopheles arabiensis* from Burkina Faso (West Africa)

**DOI:** 10.1371/journal.pone.0141645

**Published:** 2015-11-03

**Authors:** Roch K. Dabiré, Moussa Namountougou, Abdoulaye Diabaté, Dieudonné D. Soma, Joseph Bado, Hyacinthe K. Toé, Chris Bass, Patrice Combary

There is an error in the fifth sentence of the Abstract. The correct sentence is: Furthermore we report, for the first time, the identification of the *ace*.*1* G119S mutation in *An*. *arabiensis* populations collected at 8 sites.

There is also an error in the first sentence of the Discussion section. The correct sentence is: This study provides current information on the distribution of three members of the *Anopheles gambiae* complex across Burkina Faso and the frequency and distribution of three important target-site resistance mechanisms in these populations.

In Tables 2 and 3, the values in the column [95% CI] are incorrect. Please view the corrected Tables 2 and 3 here.

**Table 2:** Allelic and genotypic frequencies at the *kdr* 1014F and 1014S locus in *An*. *gambiae* s.l populations.

Species	Sites	N	Genotypes						Genotypes				
			1014L	1014L	1014F	f(L1014F)	[95%Cl]	p(HW)	1014L	1014L	f(L1014F)	[95%Cl]	p(HW)
1014L	1014F	1014F				1014L	1014F			
***An*. *arabiensis***	Gaoua	5	1	0	2	0.66	[0.24–1]	-	0	2	0.66	[0.24–1]	0.2000
	Banfora	0	0	0	0	-	-	-	0	0	0	-	-
	Sindou	10	5	0	0	0	-	-	1	4	0.9	[0.71–1]	-
	Orodara	1	1	0	0	0	-	0.4678	0	0	0	-	-
	Dioulassoba	30	1	5	14	0.82	[0.68–0.95]	0.2308	2	8	0.42	[0.24–0.59]	0.0003
	Soumousso	11	1	1	5	0.78	[0.53–1]	0.0956	2	2	0.37	[0.08–0.65]	0.2914
	Boromo	17	2	3	12	0.79	[0.59–0.98]	0.000	6	3	0.28	[0.06–0.49]	0.3405
	Dédougou	23	6	0	10	0.62	[0.42–0.81]	0.1652	5	2	0	-	0.3213
	Koudougou	13	2	3	8	0.73	[0.48–0.97]	-	0	0	0.5	[0.22–0.77]	-
	Nanoro	6	4	0	0	0	-	0.4406	0	2	0.5	[0.09–0.90]	0.0857
	Koupela	13	2	3	3	0.56	[0.29–0.82]	0.2970	2	3	0.53	[0.25–0.80]	0.1795
	Fada	25	6	5	3	0.39	[0.19–0.58]	0.0933	8	3	0.57	[0.37–0.76]	0.9035
	Kaya	17	4	3	5	0.54	[0.30–0.77]	-	1	4	0.37	[0.14–0.59]	0.0061
	Ouahigouya	1	0	1	0	0.5	[0–1]	0.0031	0	0	0	-	-
	Dori	22	6	2	8	0.56	0.35–0.76]	-	4	2	0.26	[0.07–0.44]	0.2260
***An*. *coluzzii***	Gaoua	1	1	0	0	0	-	-	0	0	0	-	-
	Banfora	7	0	1	5	0.91	[6.69–1]	-	0	1	0.16	[0–0.43]	0.0909
	Sindou	12	1	1	4	0.75	[0.50–0.99]	0.2727	1	5	0.91	[0.74–1	-
	Orodara	5	2	1	1	0.37	[0–0.79]	0.4286	1	0	0.12	[0–0.40]	-
	Dioulassoba	9	1	1	3	0.7	[0.40–0.99]	0.3333	1	3	0.7	[0.40–0.99]	0.3333
	Soumousso	4	2	1	0	0.16	[0–0.51]	-	0	1	0.33	[0–0.79]	0.2000
	Boromo	0	0	0	0	-	-	-	0	0	-	-	-
	Dédougou	3	1	1	0	0.25	[0–0.74]	-	0	1	0.5	[0–1]	0.6190
	Koudougou	7	0	3	2	0.7	|0.36–1]	1	2	0	0.2	[0–0.5]	-
	Nanoro	39	1	5	18	0.85	[0.73–0.96]	0.3983	12	3	0.37	[0.21–0.52]	0.3333
	Koupela	9	3	5	0	0.31	|0–0.61]	1	1	0	0.06	[0–0.21]	0.7446
	Fada	46	7	7	13	0.61	[0.46–0.75]	0.0186	17	2	0.38	[0.23–0.52]	0.0817
	Kaya	8	2	0	3	0.6	[0.26–0.93]	0.0476	2	1	0.4	[0.06–0.73]	0.3333
	Ouahigouya	17	4	0	6	0.6	[0.36–0.83	0.0017	2	5	0.6	[0.36–0.83]	1
	Dori	9	3	0	2	0.4	[0.07–0.72]	0.0476	1	3	0.7	[0.40–0.99]	-
***An*. *gambiae***	Gaoua	74	14	8	17	0.53	[0.41–0.64]	0.0002	0	35	0.92	[0.85–0.98]	1
	Banfora	29	7	7	10	0.56	[0.37–0.74]	0.0434	3	2	0.14	[0.01–0.26]	0.1518
	Sindou	46	8	3	13	0.6	[0.45–0.74]	0.0003	5	17	0.81	[0.69–0.92]	0.0611
	Orodara	33	5	7	11	0.63	[0.46–0.79]	0.0904	1	9	0.41	[0.24–0.57]	0.0420
	Dioulassoba	8	0	1	3	0.87	[0.63–1]	-	2	2	0.75	[0.44–1]	0.3257
	Soumousso	29	8	9	3	0.37	[0.19–0.54]	0.5690	5	4	0.32	[0.15–0.48]	0.0000
	Boromo	25	8	7	1	0.28	|0.10–0.45]	0.7912	4	5	0.43	[0.23–0.62]	0.1201
	Dédougou	19	5	0	7	0.58	[0.35–0.80]	0.0004	7	0	0.29	[0.08–0.49]	0.0150
	Koudougou	26	9	2	8	0.47	[0.27–0.66]	0.0005	4	3	0.26	[0.09–0.42]	1
	Nanoro	5	1	0	3	0.75	[0.37–1]	0.1429	0	1	0.25	[0–0.62]	0.1429
	Koupela	24	7	1	6	0.46	[0.26–0.65]	0.0013	4	6	0.57	[0.37–0.76]	0.0003
	Fada	30	3	9	7	0.6	[0.42–0.77]	0.6254	5	6	0.44	[0.26–0.61]	0.0473
	Kaya	19	5	7	3	0.43	[0.20–0.65]	0.5785	3	1	0.16	[0–0.32]	0.0000
	Ouahigouya	30	10	3	7	0.42	[0.24–0.59]	0.0020	2	8	0.45	[0.27–0.62]	0.0632
	Dori	18	4	4	4	0.5	[0.26–0.73]	0.2300	1	5	0.55	[0.32–0.77]	0.0520

N: number of mosquitoes

f(1014F): frequency of the kdr W resistant allele

f(1014S): frequency of the kdr E resistant allele

p(HW): probability of the exact test for goodness of fit to Hardy Weinberg equilibrium

-: not determined

**Table 3:** Allelic and genotypic frequencies at the ace-1 locus in *An*. *gambiae s*.*l* populations from 15 sites in Burkina Faso.

Species	Sites	N	Genotypes			f(119S)	[95%Cl]	p(HW)
			119G 119G	119G 119S	119S 119S			
*An*. *arabiensis*	Gaoua	3	3	0	0	0	-	-
	Banfora	0	0	0	0	-	-	-
	Sindou	5	5	5	0	0	-	-
	Orodara	1	1	1	0	0	-	-
	Dioulassoba	20	4	4	12	0.7	[0.49–0.90]	0.0264
	Soumousso	7	1	1	5	0.78	[0.47–1]	0.2308
	Boromo	15	5	9	1	0.36	[0.11–0.60]	0.9488
	Dédougou	14	4	6	4	0.5	[0.23–0.76]	0.0444
	Koudougou	12	5	0	7	0.58	[0.30–0.85]	0.0004
	Nanoro	3	2	0	1	0.33	[0–0.86]	0.2000
	Koupela	8	8	0	0	0	-	-
	Fada	13	4	8	1	0.38	[0.11–0.64]	0.9449
	Kaya	12	11	0	1	0.08	[0–0.23]	0.0435
	Ouahigouya	1	1	0	0	0	-	-
	Dori	14	14	0	0	0	-	-
*An*. *coluzzii*	Gaoua	1	1	0	0	0	-	-
	Banfora	6	6	0	0	0	-	-
	Sindou	6	6	0	0	0	-	-
	Orodara	4	4	0	0	0	-	-
	Dioulassoba	5	4	1	0	0.1	[0–0.36]	-
	Soumousso	3	3	0	0	0	-	-
	Boromo	0	0	0	0	-	-	-
	Dédougou	2	0	0	2	0.5	[0–1]	1
	Koudougou	5	2	3	0	0.3	[0–0.70]	1
	Nanoro	23	17	6	0	0.13	[0–0.26]	1
	Koupela	8	6	2	0	0.12	[0–0.34]	1
	Fada	27	27	0	0	0	-	-
	Kaya	5	5	0	0	0	-	-
	Ouahigouya	9	6	3	0	0.16	[0–0.39]	1
	Dori	5	5	0	0	0	-	-
*An*. *gambiae*	Gaoua	36	22	11	3	0.23	[0.09–0.36]	0.2811
	Banfora	24	20	4	0	0.08	[0–0.18]	1
	Sindou	24	21	3	0	0.06	[0–0.15]	1
	Orodara	23	22	0	1	0.04	[0–0.12]	0.0222
	Dioulassoba	4	4	0	0	0	-	-
	Soumousso	20	18	0	2	0.1	[0–0.23]	0.0021
	Boromo	15	9	4	2	0.26	[0.03–0.48]	0.2260
	Dédougou	12	8	4	0	0.16	[0–0.36]	1
	Koudougou	18	14	1	3	0.19	[0–0.37]	0.0029-
	Nanoro	4	3	1	0	0.12	[0–0.43]	-
	Koupela	12	12	0	0	0	-	-
	Fada	19	18	0	1	0.05	[0–14]	0.0270
	Kaya	15	11	4	0	0.13	[0–0.30]	1
	Ouahigouya	19	14	2	3	0.21	[0.02–0.39]	0.0096
	Dori	11	10	0	1	0.09	[0–0.25]	0.0476

N: number of mosquitoes

f(119S): frequency of the 119S resistant ace.1 allele

p(HW): probability of the exact test for goodness of fit to Hardy Weinberg equilibrium

-: not determined
